# The Mascarene Archipelago, a Data‐Poor Region for Mobulid Rays: First Records, Seasonal Patterns and Conservation Implications

**DOI:** 10.1002/ece3.73815

**Published:** 2026-06-23

**Authors:** Joanna L. Harris, Estelle Crochelet, Prashant Mohesh, Guy M. W. Stevens

**Affiliations:** ^1^ The Manta Trust Dorset UK; ^2^ School of Biological and Marine Sciences University of Plymouth Plymouth UK; ^3^ Agence de Recherche Pour la Biodiversité à La Réunion, Biodiversity Research Agency of Reunion Island, Mascarene Archipelago Elasmobranch Observatory La Réunion France; ^4^ The National Geographic Society (Mauritius) Washington DC USA

**Keywords:** citizen science, elasmobranchs, Mascarene plateau, seasonal aggregation, species distribution, Western Indian Ocean

## Abstract

The distribution and seasonal occurrence of manta and devil rays (mobulids) remain poorly understood across much of the Western Indian Ocean, particularly within remote island systems. Here, we present the first synthesis of species‐level mobulid records for the Mascarene Archipelago (La Réunion, Mauritius and Rodrigues) using a combination of direct and passive citizen‐science observations collected between 2014 and 2026. A total of 107 sightings were documented from 75 sighting events, comprising *Mobula birostris* (*n* = 29), *M. thurstoni* (*n* = 40), *M. alfredi* (*n* = 5), *M. tarapacana* (*n* = 2) and unidentified *Mobula* spp. (*n* = 31). One *M. birostris* was re‐sighted almost 4 years after it was first recorded (October 2019 to September 2023). Records were strongly concentrated along the west–south‐west coast of La Réunion, accounting for 90% of all sightings, whereas confirmed sightings in Mauritius were fewer, and no records were obtained for Rodrigues. We report the first confirmed records of *M. birostris* and *M. tarapacana* in Mauritian and La Réunion waters, respectively. Circular analyses revealed seasonal occurrences for the most frequently recorded species, with peak occurrence during late austral spring to early summer. Courtship behaviour for *M. thurstoni* is rarely observed globally; thus, these findings demonstrate that the Mascarene Archipelago supports multiple mobulid species and is used for ecologically important activities, suggesting that the region forms part of a broader, seasonally structured habitat network associated with the Mascarene Plateau. In the context of the recent transfer of all mobulids to CITES Appendix I and ongoing regional fisheries interactions, this baseline highlights the value of citizen‐science data for identifying mobulid presence in data‐poor regions and provides a foundation for targeted monitoring and conservation planning in the Western Indian Ocean.

## Graphical abstract

This study provides the first synthesis of mobulid ray occurrence in the Mascarene Archipelago, using citizen science data to document species diversity, spatial distribution and seasonal patterns, including the first confirmed records of Mobula birostris in Mauritius and *M. tarapacana* in La Réunion. It reveals that multiple species utilise the region, with strong seasonal peaks in late austral spring to early summer and evidence of ecologically important behaviours, including rare courtship observations. These findings highlight the archipelago as a previously under‐recognised, seasonally important habitat and demonstrate the value of opportunistic data for informing conservation and management in data‐poor regions.

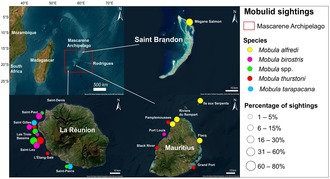

## 1 Introduction

Manta and devil rays, collectively known as mobulids, are filter‐feeding elasmobranchs comprising 10 species distributed across tropical and subtropical oceans (Marshall et al. 2009; White et al. 2017; Hosegood et al. 2020; Bucair et al. 2025). Mobulids are fished for their cartilaginous gill plates and meat (Palacios et al. 2025). These targeted fisheries, combined with slow growth and low reproductive rates, have led to substantial population declines (Laglbauer et al. 2026). Compounding the threats from fisheries are human‐induced climate change, habitat degradation, pollution and unregulated tourism (Dulvy, Fowler, et al. 2014; Williams et al. 2018; Strike et al. 2022). Consequently, all mobulid species are listed as Vulnerable to Critically Endangered on the IUCN Red List of Threatened Species (IUCN 2025), in Appendices I and II of the Convention on the Conservation of Migratory Species of Wild Animals (CMS 2017) and in Appendix I of the Convention on International Trade in Endangered Species (CITES 2025).

Mobulids are highly mobile, exhibiting a wide range of movement strategies that span high site fidelity to large‐scale, seasonal movements that can cross ocean basins (Couturier et al. 2012; Francis and Jones 2017; Mendonça et al. 2018; Harris, Mcgregor, et al. 2020; Palacios et al. 2023; Harris, Hosegood, et al. 2024; Stevens et al. 2025). Using methods such as visual surveys and acoustic and satellite telemetry, seasonal occurrence patterns have been documented for multiple mobulid species and are often linked to changes in oceanographic conditions that influence prey availability, such as monsoon systems, climatic patterns, upwelling, mesoscale eddies, sea surface temperature and current–bathymetry interactions (Lezama‐Ochoa et al. 2019; Harris, Mcgregor, et al. 2020; Armstrong et al. 2021; Harris et al. 2021; di Sciara et al. 2025).

Citizen science has played an integral role in the collection of data for many studies aiming to advance our understanding of mobulid spatial ecology (e.g., Beard et al. 2021; Ehemann et al. 2022; Pate et al. 2025). For species with stable, individually distinctive ventral markings, such as the reef (*Mobula alfredi*), oceanic ( *M. birostris* ) and Atlantic (*M. yarae*) manta rays and the sicklefin devil ray ( *M. tarapacana* ), photographic identification (photo‐ID) has enabled the delineation of populations, estimation of population size and assessment of spatial connectivity and site fidelity (Armstrong et al. 2019; Harris, Mcgregor, et al. 2020; Harris and Stevens 2021; Rambahiniarison et al. 2022; Pate et al. 2025). Nonetheless, critical gaps remain regarding the full extent of mobulid ranges and distributions, particularly within remote island systems where monitoring can be logistically challenging (Stewart et al. 2018; Notarbartolo Di Sciara et al. 2020; Harris and Stevens 2024; Stevens et al. 2025). These gaps limit the development of conservation and management strategies that adequately reflect the spatial and temporal dynamics of these highly mobile rays (Lawson et al. 2017; Stewart et al. 2018).

**FIGURE 1:**
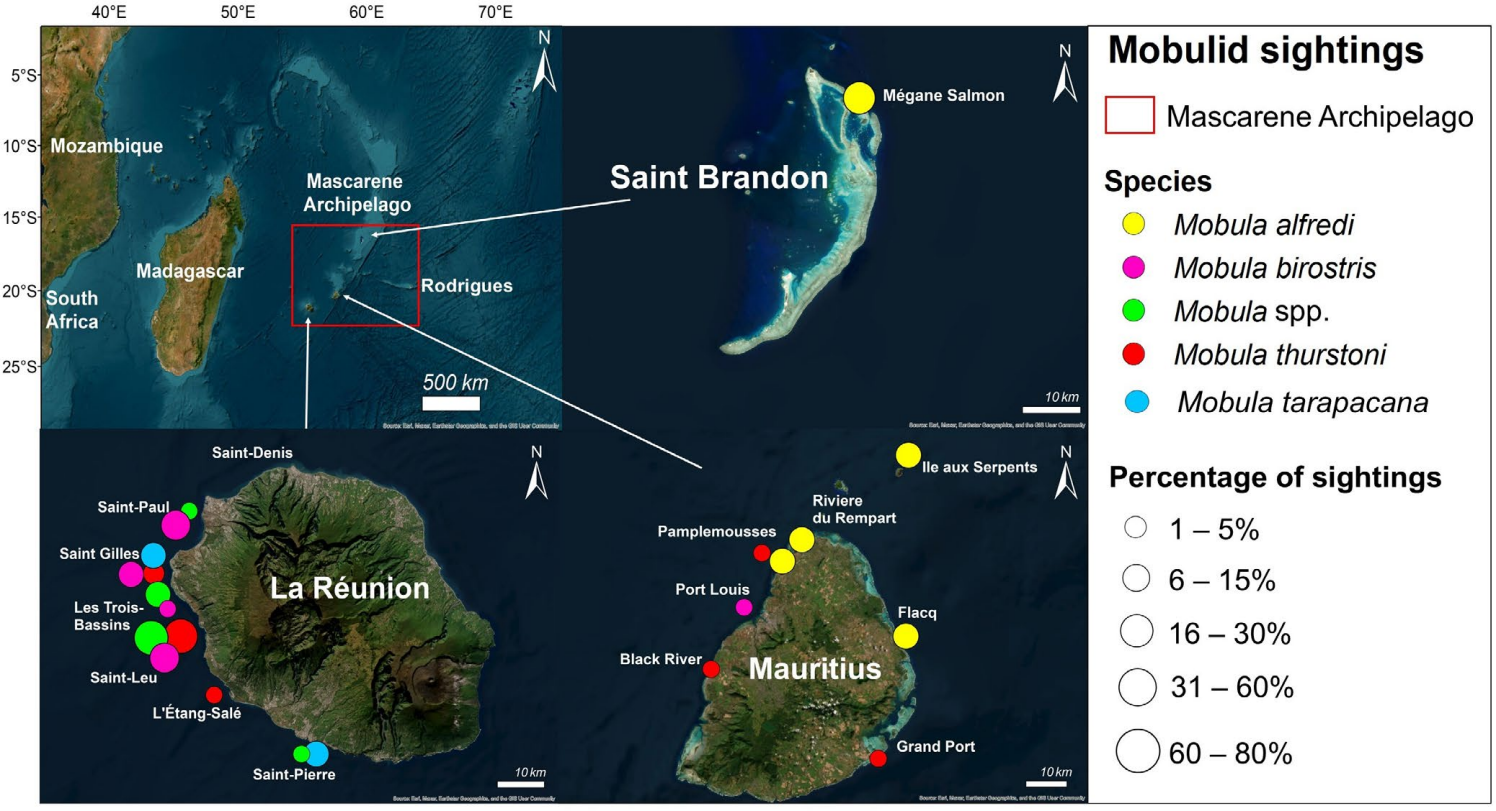
The Mascarene Archipelago in the western Indian Ocean (top‐left panel) shows the spatial distribution of mobulid sightings across three islands (La Réunion, Mauritius and Saint Brandon, an archipelago belonging to Mauritius) between 2014 and 2026 (remaining panels). Coloured symbols indicate species identity: *Mobula alfredi* (*n* = 5), *M. birostris* (*n* = 29), *M. thurstoni* (*n* = 40), *M. tarapacana* (*n* = 2) and unidentified *Mobula* spp. (*n* = 31). Symbol size represents the percentage of the total number of sightings of each observed species across the Mascarene Archipelago. Rodrigues is not shown, as no mobulid sightings were documented at this location. For visual clarity, sightings were grouped by community (Table 1) and where multiple species occurred at the same location, points were jittered.

The Mascarene Archipelago (La Réunion, Mauritius, Rodrigues; Figure 1 and Table 1) is a remote island system situated on the Mascarene Plateau in the Western Indian Ocean, where seasonal upwelling and nutrient enrichment enhance regional productivity and biodiversity (Myers et al. 2000; Gallienne et al. 2004). Along with productive upwelling, these islands have extensive reef systems and seamount complexes (Turner and Klaus 2005) similar to those that support mobulid populations in other areas of the Indian Ocean (Harris, Mcgregor, et al. 2020; Harris, Hosegood, et al. 2024). However, mobulid research in the Mascarene Archipelago has been limited, with few published works on the presence of mobulid species (Letourneur et al. 2004; Crochelet et al. 2025). Here, we harness direct and passive citizen science records to synthesise confirmed species‐level mobulid sightings in the Mascarene Archipelago, quantify their spatial distribution and test for seasonal clustering, providing a baseline for future monitoring and highlighting the value of opportunistic data for conservation.

**TABLE 1:** Percentage of sightings of each mobulid species by country, community and location (Figure 1).

Country	Community	Location	Latitude	Longitude	*Mobula alfredi*	*Mobula birostris*	*Mobula* spp.	*Mobula tarapacana*	*Mobula thurstoni*
Mauritius	Belle Mare	Belle Mare	−20.188	57.804	20% (*n* = 1)	—	—	—	—
Mauritius	Ile aux Serpents	Ile aux Serpents	−19.816	57.809	20% (*n* = 1)	—	—	—	—
Mauritius	La Mecque	La Mecque	−20.256	57.377	—	—	—	—	3% (*n* = 1)
Mauritius	Pereybere	Pereybere	−19.990	57.576	20% (*n* = 1)	—	—	—	—
Mauritius	Pointe d'Esny	Pointe d'Esny	−20.440	57.743	—	—	—	—	5% (*n* = 2)
Mauritius	Port Louis	Port Louis	−20.120	57.407	—	3% (*n* = 1)	—	—	—
Mauritius	Saint Brandon	Saint Brandon	−16.466	59.682	20% (*n* = 1)	—	—	—	—
Mauritius	Trou aux Biches	Trou aux Biches	−20.034	57.533	20% (*n* = 1)	—	—	—	5% (*n* = 2)
Réunion	Les Trois‐Bassins	Passe des Trois‐Bassins	−21.129	55.266	—	3% (*n* = 1)	—	—	—
Réunion	L'Étang‐Salé	L'Étang‐Salé	−21.283	55.341	—	—	—	—	3% (*n* = 1)
Réunion	Saint Gilles	Boucan Canot	−21.026	55.224	—	7% (*n* = 2)	19% (*n* = 6)	—	8% (*n* = 3)
Réunion	Saint Gilles	Passe de l'Hermitage	−21.086	55.223	—	10% (*n* = 3)	3% (*n* = 1)	—	—
Réunion	Saint Gilles	Saint‐Gilles	−21.053	55.221	—	7% (*n* = 2)	—	50% (*n* = 1)	—
Réunion	Saint‐Leu	Pointe au Sel	−21.205	55.279	—	17% (*n* = 5)	71% (*n* = 22)	—	75% (*n* = 30)
Réunion	Saint‐Leu	Pointe des Châteaux	−21.149	55.270	—	3% (*n* = 1)	—	—	—
Réunion	Saint‐Leu	Saint‐Leu	−21.172	55.283	—	14% (*n* = 4)	—	—	3% (*n* = 1)
Réunion	Saint‐Paul	Baie de Saint‐Paul	−21.011	55.250	—	7% (*n* = 2)	3% (*n* = 1)	—	—
Réunion	Saint‐Paul	Cap La Houssaye	−21.017	55.236	—	28% (*n* = 8)	—	—	—
Réunion	Saint‐Pierre	Grand Bois	−21.355	55.506	—	—	3% (*n* = 1)	—	—
Réunion	Saint‐Pierre	Rivière Sainte Etienne	−21.332	55.372	—	—	—	50% (*n* = 1)	—

## 2 Materials and Methods

### 2.1 Data Collection

In the context of this study, a sighting event is defined as a confirmed observation of one or more mobulid rays occurring simultaneously on a given day at a defined location. Each individual observed within a sighting event was treated as a separate sighting. All species identifications were verified by the authors from morphological characteristics (Stevens et al. 2025). Uncertain identifications were assigned to *Mobula* spp. All images showing the ventral spots and patterning of the relevant species were checked using the Manta Trust's MantaBase and IDtheManta tools (https://mantabase.org/home) to identify matches, and the results were verified visually by trained experts.

Mobulid sighting records were obtained through submissions to the Citizen Science Platform (www.reseau.maeoproject.org) and passive citizen science (Edwards et al. 2021). The Citizen Science Platform was established in 2021 as part of the Mascarene Archipelago Elasmobranch Observatory (MAEO) and gives users (e.g., divers, snorkellers, fishers) the option to submit sightings with photos or videos and associated metadata, including date, location, depth, number of individuals, sex and behavioural activity. Passive citizen science sightings were obtained through online Google searches of publicly available images and videos shared on social media platforms (Facebook, Instagram, YouTube and Flickr) using the following Boolean search string: (‘mobulid’ OR ‘manta’ OR ‘devil ray’ OR ‘*Mobula alfredi*’ OR ‘reef manta ray’ OR ‘*Mobula birostris*’ OR ‘oceanic manta’ OR ‘ *Mobula tarapacana* ’ OR ‘sicklefin devil ray’ OR ‘ *Mobula thurstoni* ’ OR ‘bentfin devil ray’ OR ‘ *Mobula mobular* ’ OR ‘spinetail devil ray’) + (‘Mascarene’ OR ‘La Réunion’ OR ‘Mauritius’ OR ‘Rodrigues’). Separate searches were conducted for each media platform by adding site:facebook.com, site:Instagram.com, site:youtube.com, or site:flickr.com to the start of each search string. Searches included only the mobulid species expected to inhabit the Mascarene Archipelago (Stevens et al. 2025).

### 2.2 Mapping

For each mobulid species, the percentage of sightings recorded at each location was calculated relative to the total number of sightings observed across the Mascarene Archipelago and projected in ArcMap 10.8.1.

### 2.3 Seasonal Analysis

Seasonal patterns in mobulid occurrence were assessed using circular statistics, which are appropriate for analysing periodic data such as the month of observation (Pewsey et al. 2013). All analyses were conducted in R version 4.4.2 (R Core Team 2023). Dates of mobulid sightings were converted to angular data by assigning each observation to the midpoint of its calendar month (15th day) and transforming the corresponding day‐of‐year to radians (0–2π). To account for variation in group size among sightings, analyses were weighted by the number of individuals observed per record.

Seasonal clustering in mobulid occurrence was tested using the Rayleigh test for circular uniformity, which evaluates whether observations are evenly distributed throughout the year or concentrated around a single seasonal peak (Pewsey et al. 2013). The strength of seasonal clustering was quantified using the mean resultant length (r), with values approaching zero indicating a uniform distribution and values approaching one indicating strong concentration around the mean direction. The mean circular direction was calculated to estimate the central timing of peak seasonal occurrence and is reported as an approximate calendar date for interpretability.

To explore potential interspecific differences in seasonal occurrence, the same circular analytical framework was applied separately to each mobulid species with sufficient sample size. To account for multiple species‐level tests, Rayleigh test *p*‐values were adjusted using a false discovery rate (FDR) correction following the Benjamini–Hochberg procedure (Benjamini and Hochberg 1995). All results were interpreted as indicative of relative seasonal occurrence patterns rather than absolute abundance, as the data were derived from opportunistic sightings and do not account for spatial or temporal variation in observation effort.

## 3 Results

**FIGURE 2:**
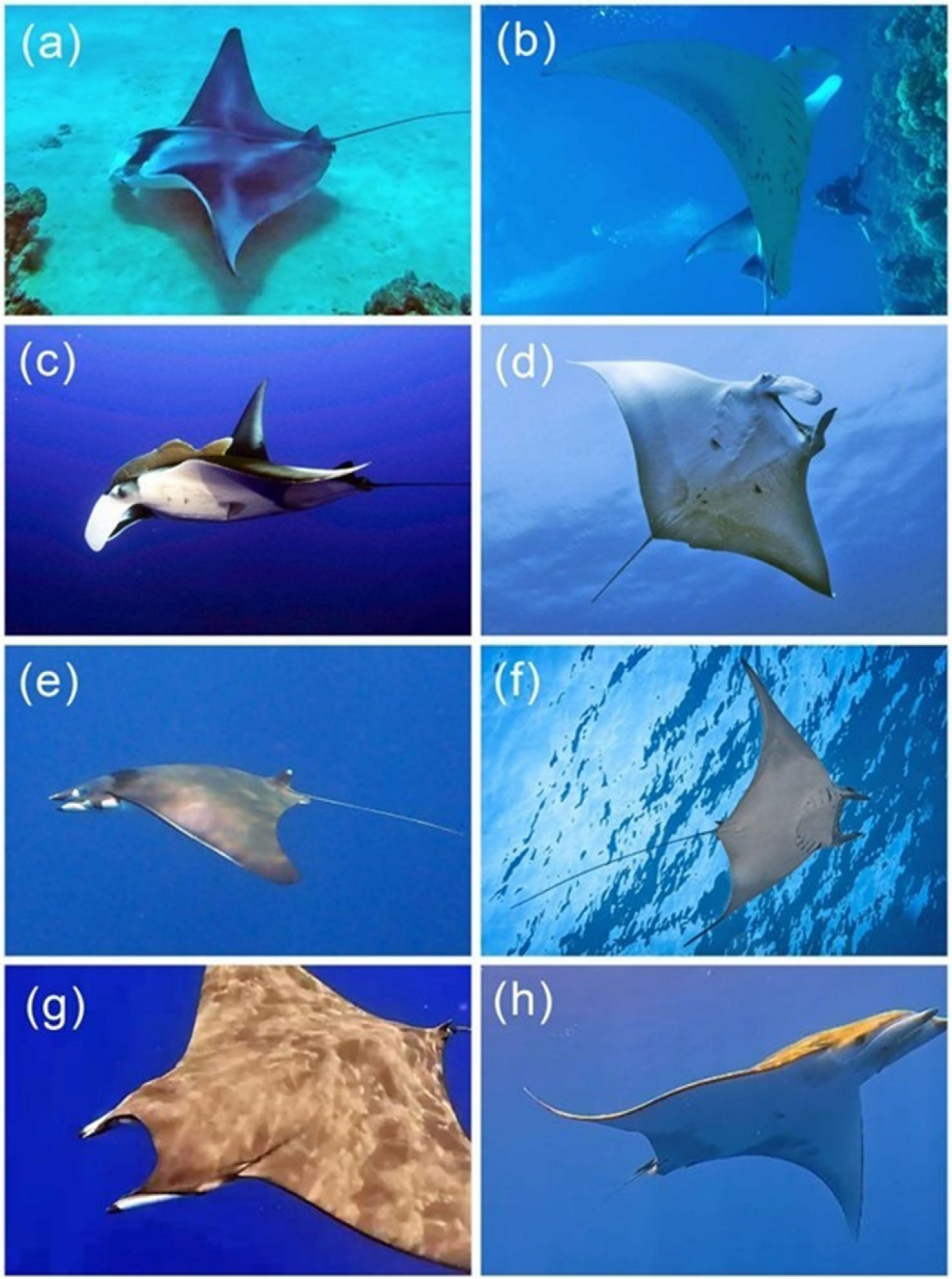
Mobulids observed in the Mascarene Archipelago: (a, b) *Mobula alfredi* encountered feeding at Trou aux Biches, Mauritius (photo credit: Gideon Malan), (c, d) *Mobula birostris* encountered at Pointe au Sel, La Réunion (photo credits: Cédric Péneau and Plongée Péï), (e, f) *M. thurstoni* encountered at Pointe au Sel, La Réunion (photo credits: Bleu Océanin and Mathieu Gatinaud) and (g, h) *M. tarapacana* encountered at Rivière Sainte Etienne, La Réunion (photo credit: Hendrik Harms).

A total of 107 mobulid sightings were recorded across 75 sighting events in the Mascarene Archipelago between 2014 and 2026. These comprised 29 oceanic manta rays (*Mobula birostris*), 40 bentfin devil rays ( *M. thurstoni* ), five reef manta rays ( *M. alfredi* ), two sicklefin devil rays ( *M. tarapacana* ) (Figure 2), and 31 individuals that could not be identified to species level (*Mobula* spp.). Of the 107 sightings recorded, 96 (90%) were observed on the west–south‐west coast of La Réunion, including *M. birostris* (*n* = 29), *M. thurstoni* (*n* = 35), *M. tarapacana* (*n* = 2) and unidentified mobulids (*Mobula* spp.; *n* = 31). Two *M. birostris* sightings in La Réunion were confirmed to be the same individual based on their unique ventral spot patterns. These sightings occurred in October 2019 and September 2023. All sightings recorded in Mauritius were identified to the species level and comprised *M. alfredi* (*n* = 5), *M. birostris* (*n* = 1) and *M. thurstoni* (*n* = 5). No mobulid sightings were documented for Rodrigues.

Behaviour was reported by observers for 37 of the 75 sighting events. Of these, 33 encounters were described as cruising, including observations of *M. alfredi* (*n* = 3), *M. birostris* (*n* = 15), *Mobula* spp. (*n* = 1) and *M. thurstoni* (*n* = 14). Courtship behaviour was recorded for *M. thurstoni* on two occasions, both at Pointe au Sel, La Réunion, in November 2021 and December 2025. Feeding behaviour was recorded on two occasions: one *M. birostris* observed feeding at Baie de Saint‐Paul, La Réunion, in October 2023, and one *M. alfredi* observed feeding at Trou aux Biches, Mauritius, in January 2025.

### 3.1 Seasonal Analysis

**FIGURE 3:**
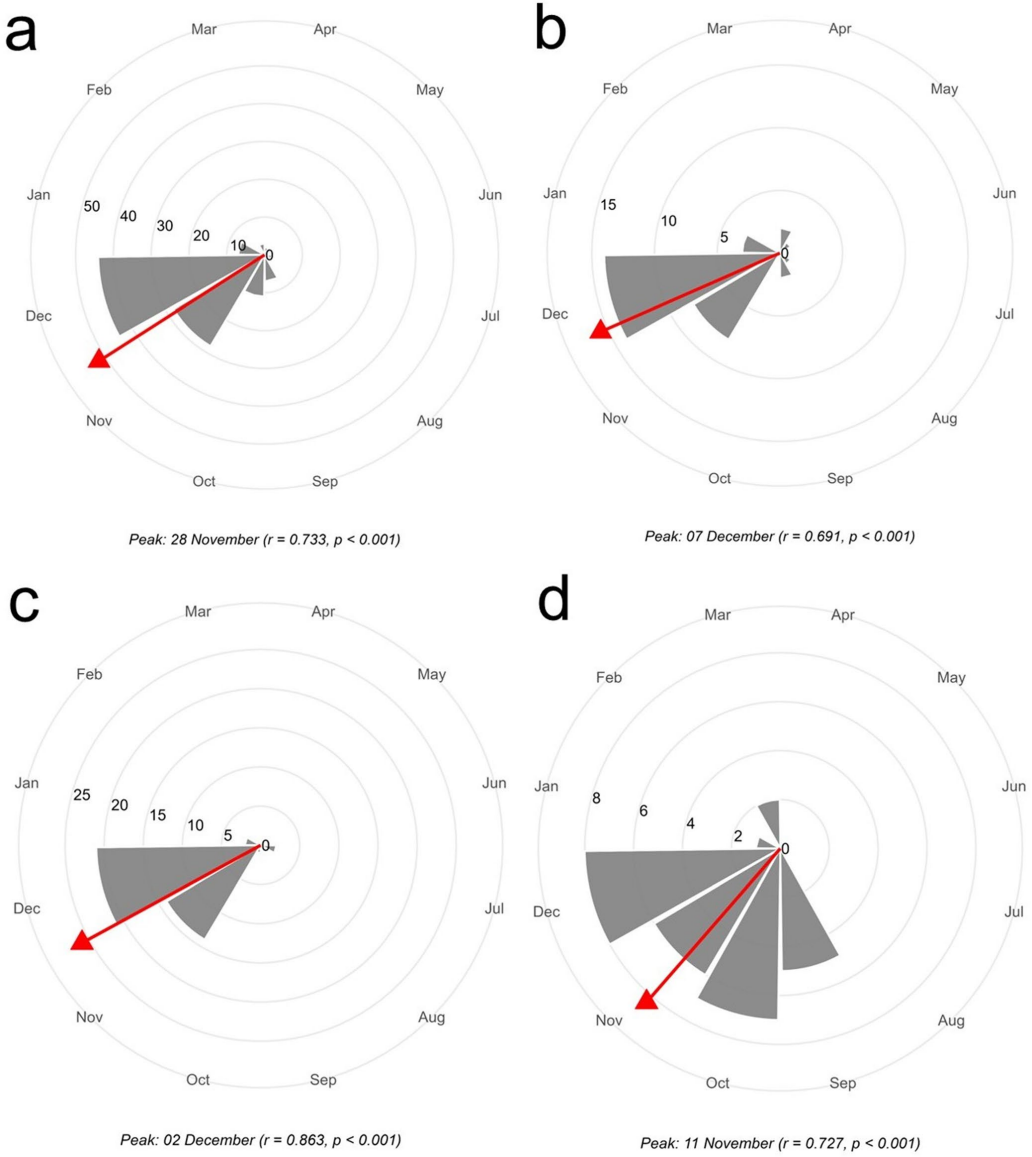
Seasonal distribution of mobulid sightings in the Mascarene Archipelago. Panels show monthly occurrence for (a) all mobulids combined, (b) *Mobula* spp., (c) *M. thurstoni* and (d) *M. birostris* . *Mobula alfredi* is not shown as they had insufficient data for circular inference. Grey bars indicate the total number of individuals observed per month, summed across records and weighted by group size. Angular position represents the month of observation and radial distance (concentric rings) represents the number of individuals. Red arrows indicate the circular mean timing of peak seasonal occurrence derived from circular statistics, with associated mean resultant length (r) and Rayleigh test *p*‐values shown where sample size was sufficient. Results represent relative seasonal occurrence and are not corrected for observation effort.

Mobulid sightings were seasonal and significantly clustered throughout the years (Rayleigh test for circular uniformity: Z = 0.73, *p* < 0.001), with a mean seasonal peak centred in late November (mean resultant length *r* = 0.73) (Figure 3a).

Species‐specific circular analyses revealed pronounced seasonal occurrence for two identified mobulid species and *M*. spp. Sightings of *M. thurstoni* (Figure 3c) were strongly clustered in early December (mean peak: 2 December; mean resultant length *r* = 0.86), with a significant departure from uniformity (Rayleigh test: *p* < 0.001). *Mobula birostris* also exhibited seasonal clustering, peaking on 11 November (*r* = 0.73; *p* < 0.001; Figure 3d), while unidentified mobulid records peaked in early December (mean peak 7 December; *r* = 0.69; *p* < 0.001; Figure 3b). Sample size was insufficient to assess *M. alfredi* and *M. tarapacana* .

## 4 Discussion

This study provides the first synthesis of species‐level mobulid occurrence in the Mascarene Archipelago and reveals that the region supports multiple mobulid species, with records dominated by *Mobula birostris* and *M. thurstoni* and more limited observations of *M. alfredi* and *M. tarapacana* . All *M. alfredi* sightings occurred in Mauritius, while most *M. birostris* sightings occurred in La Reunion. This may be consistent with a lack of suitable habitat for *M. alfredi* around La Reunion, and while there does not appear to be a resident population of *M. alfredi* in Mauritius, sightings possibly result from transient individuals moving south along the Mascarene Plateau from known source populations in Seychelles. Furthermore, the absence of *M. mobular* sightings is surprising, given their distribution and propensity to occur in habitats similar to those of the other mobulids recorded here (Stevens et al. 2025).

The strong spatial concentration of mobulid sightings along the western coast of La Réunion and the marked seasonal signal, with peak occurrence in late austral spring to early summer, indicate that mobulid presence in the Mascarenes is structured rather than incidental. Seasonal variation in mobulid presence around the Mascarenes may be linked to physical and biological processes associated with nearby landmasses, seamounts and the Mascarene Plateau. For example, it has been demonstrated that mesoscale eddies, particularly the dipole systems common from late October to December, enhance retention of ichthyoplankton and zooplankton in the region, increasing prey availability (Harris, Noyon, et al. 2020). Cross‐shelf transport from Madagascar further contributes neritic larvae offshore, while local seamount effects and water mass structure promote aggregation of planktonic communities (Harris, Noyon, et al. 2020). Furthermore, habitat suitability models suggest the Mascarene Plateau is a region of concentrated devil ray occurrence that appear to contract tightly around the Plateau during the summer months (June–September), before expanding into wider areas of the Western Indian Ocean, suggesting that the Plateau may act as a migratory corridor linking regional foraging grounds (Guirhem et al. 2021). Given that La Réunion, Mauritius and Rodrigues lie along the western and eastern margins of this Plateau, it is plausible that mobulids utilising the Mascarene Plateau also transit through or aggregate around the islands during seasonal movements. This is consistent with higher mobulid occurrence in the Mascarenes during November and December, coinciding with a period when mesoscale eddies and cross‐shelf transport enhance local prey availability (Harris, Noyon, et al. 2020). These observations suggest that the Mascarenes may serve as a seasonal aggregation sites within a broader regional movement network, which requires confirmation through telemetry or photo‐ID connectivity studies.

These seasonal patterns, together with the observation of courtship behaviour in *M. thurstoni*, a behaviour rarely observed (Mccallister et al. 2020; Palacios et al. 2024), demonstrate that the archipelago is used for ecologically significant activities. Here, we also report the first confirmed records of *M. birostris* and *M. tarapacana* in Mauritian and La Réunion waters, respectively, thereby advancing our understanding of the broader distribution of mobulids in the Western Indian Ocean. The absence of records from Rodrigues likely reflects a lack of reported sightings rather than confirmed absence, highlighting the limitations of opportunistic data and the need for targeted monitoring in under‐sampled areas.

The seasonal and spatial patterns reported here should be interpreted in the context of the opportunistic sampling design, as sighting frequency can be influenced by variation in observation effort associated with tourism; therefore, the significance of these results could reflect seasonal human behaviour and weather patterns rather than true ecological distribution. However, recreational diving occurs year‐round in La Réunion and Mauritius, indicating that the temporal signal is not driven solely by observer presence. In contrast, the pronounced concentration of records along the western coast of La Réunion reflects greater accessibility, as the eastern coastline is persistently exposed to trade winds and higher wave energy, which limits in‐water activities (Bourmaud et al. 2005). The spatial distribution, therefore, represents relative occurrence within regularly surveyed areas rather than habitat preference or absence from unsampled regions. Nevertheless, the recurrent timing of sightings and the co‐occurrence of biologically relevant behaviours support an ecological basis for the seasonal signal.

The apparent evidence of seasonal occurrence and potential migratory connectivity around the Mascarenes Archipelago highlights the need to integrate mobulids into regional conservation frameworks. La Réunion and Mauritius are signatories to CITES and CMS and members of the Indian Ocean Tuna Commission (IOTC), and therefore have a responsibility to regulate trade, protect habitats and ensure compliance with IOTC measures such as mobulid retention bans and safe handling and release guidelines (CMS 2017; IOTC 2019; CITES 2025). However, the effectiveness of these instruments depends on strong national implementation (Crochelet et al. 2025). While Mauritius has introduced a ban on shark and ray fishing within its territorial waters, and La Réunion benefits from both French and European Union regulations, enforcement gaps and a lack of systematic monitoring and reporting of mobulid interactions remain (Crochelet et al. 2025). Strengthening protections will therefore require coordinated action at both national and regional scales. For example, enhancing compliance monitoring, improving transboundary cooperation, introducing mobulid monitoring programmes and aligning local data collection with international commitments would provide a stronger evidence base for management. Furthermore, integrating local initiatives, including citizen science, community engagement and educational programmes, into policy frameworks would provide a multi‐layered approach that would enhance the development of effective mobulid conservation strategies in the Mascarenes Archipelago.

The development of regional conservation frameworks is particularly timely given the ongoing negotiations concerning the future governance of the Chagos Archipelago, which supports important local populations of several mobulid species (Harris, Hosegood, et al. 2024; Harris, Collins, et al. 2024) that may in future fall under Mauritian management. The archipelago is currently encompassed by one of the world's largest no‐take marine protected areas (MPA) (Sheppard et al. 2012; Hays et al. 2020), and its management framework may evolve under Mauritian jurisdiction, for example, through spatial zoning that permits fishing or tourism activities. The effectiveness of the MPA will depend on strong species‐level protection and effective enforcement under any future governance arrangement. The islands of Chagos were formerly inhabited by the Chagossians, and accounts of Chagossian cultural memory describe mobulids as part of their fishery (Patel 2019). This underscores both the cultural significance of these species and the importance of inclusive, socially equitable management under any future resettlement scenario. Given the extremely low productivity and high extinction risk of mobulids, even low levels of mortality are not biologically sustainable (Dulvy, Pardo, et al. 2014; Barrowclift et al. 2025; Laglbauer et al. 2026). Ensuring their long‐term survival will therefore require retention bans and species‐level legal protections across all fisheries, including small‐scale and subsistence sectors, supported by effective monitoring, surveillance and regional cooperation.

## Author Contributions

**Joanna L. Harris:** data curation (equal), formal analysis (lead), investigation (supporting), methodology (lead), writing – original draft (lead), writing – review and editing (equal). **Estelle Crochelet:** data curation (equal), investigation (equal), writing – review and editing (equal). **Prashant Mohesh:** investigation (supporting), writing – review and editing (equal). **Guy M. W. Stevens:** data curation (equal), investigation (equal), supervision (lead), writing – review and editing (equal).

## Funding

The authors have nothing to report.

## Conflicts of Interest

The authors declare no conflicts of interest.

## Data Availability

Data is available on request from the Mascarene Archipelago Elasmobranch Observatory (MAEO) and the Manta Trust's MantaBase platform (https://mantabase.org/home). It is also available from Figshare (https://figshare.com/s/6f650cf06f5ef9f17901).
